# Can multisite clinical trial results change clinical practice? Use of long-acting injectable risperidone nationally in the Veterans Health Administration

**DOI:** 10.1186/s13063-023-07094-6

**Published:** 2023-02-06

**Authors:** Robert Rosenheck, Sonia T. Anand, Stephen G. Kurtz, Cynthia Hau, Diane Smedberg, James F. Pontzer, Ryan E. Ferguson, Cynthia R. Davis

**Affiliations:** 1 VA New England Mental Illness, Research, Education and Clinical Center, 151D, 950 Campbell Ave., West Haven, CT 06516 USA; 2 Department of Psychiatry, Yale Medical School, West Haven, CT USA; 3 VA Cooperative Studies Program Coordinating Center, Boston, MA USA; 4 VA Clinical Research Pharmacy Coordinating Center, Albuquerque, NM USA; 5 VA Office of Research and Development, Cooperative Studies Program, Albuquerque, NM USA; 6 Boston University School of Medicine, Boston, MA USA; 7 Harvard Medical School, Boston, MA USA

**Keywords:** Clinical trial impact, Long-acting antipsychotic medication, Schizophrenia, Dissemination of research

## Abstract

**Background:**

Multisite practical clinical trials evaluate treatments in real-world practice. A multisite randomized Veterans Health Administration (VHA) cooperative study (CSP#555) published in 2011 compared the first long-acting injectable (LAI) second-generation antipsychotic (SGA), Risperidone Consta®, in veterans with a diagnosis of schizophrenia or schizoaffective disorder, to oral antipsychotics, with unexpected null results for effectiveness and cost-effectiveness. Whether null results of this type could change VHA practice has not been studied.

**Methods:**

A longitudinal observational analysis was used to evaluate the impact of the trial findings on VHA clinical practices. National administrative data compared new starts on LAI risperidone during the 4 years before the publication of CSP#555 in 2011 to new starts on LAI risperidone during the 4 years after.

**Results:**

Among 119,565 Veterans with the indicated diagnoses treated with antipsychotics from 2007 to 2015, the number and proportion of new starts on LAI risperidone declined significantly following the study publication, as did the total number of annual users and drug expenditures. However, data from 2007 to 2010 showed the decline in new starts actually preceded the publication of CSP#555. This change was likely explained by the increase in new starts, total use, and expenditures on a newer medicine, LAI paliperidone, a 4-week LAI treatment, in the 2 years *prior* to the publication of CSP#555.

**Conclusions:**

The declining use of LAI risperidone likely primarily reflects the substitution of a longer-acting LAI SGA, paliperidone, that came to market 2 years *before* the study publication, a substitution that may have been reinforced by null CSP#555 study results for LAI risperidone.

## Background

Short-term placebo-controlled randomized clinical trials (RCTs), conducted to obtain Food and Drug Administration (FDA) approval for commercial marketing of new drugs, do not provide adequate information for determining whether the longer-term effectiveness and safety of such medications merit widespread replacement of older, often less expensive, drugs already on the market [1]. Practical clinical trials or comparative effectiveness studies compare new drugs to widely used comparators at multiple sites, under longer-term, real-world clinical treatment conditions, with minimal exclusion criteria. These studies have become recognized as critical for the evaluation of health benefits of new medications and for fostering their widespread use [2, 3].

Since the 1940s, the Veterans Health Administration (VHA), the largest healthcare system in the USA, has committed itself to the conduct of such trials through its Cooperative Studies Program (CSP) which maintains a unique infrastructure of coordinating centers within the VHA to support comparative effectiveness research across more than 150 Veterans Affairs (VA) medical centers nationwide for the specific purpose of improving treatment in VHA [4]. The program has been responsible for landmark studies of antibiotics for tuberculosis, antihypertensive medications, antipsychotics for schizophrenia, and many other major studies to “produce innovative and effective solutions to Veteran and national healthcare problems” [4].

In spite of this clear objective, there has been limited evaluation of the impact of individual VA cooperative studies on clinical practice in the VA healthcare system. There has long been concern about the often prolonged gap between published scientific findings and changes in clinical practice [5, 6]. Limited attention has been paid to the naturalistic dissemination of trial findings, such as those from the VA CSP, into clinical practice, especially in the healthcare systems that sponsored the research and in which it was conducted.

Schizophrenia, the focus of this study, is perhaps the most disabling of psychiatric conditions affecting approximately 1% of all adults and almost 100,000 VA patients each year at a cost of thousands of dollars per patient [7, 8]. A major challenge in the treatment of schizophrenia is that many patients lack insight into their condition and are inconsistent in their use of prescribed medications or refuse them, leading to recidivism and costly rehospitalization [9, 10]. One tool to improve medication adherence in the treatment of schizophrenia is the use of long-acting injectable (LAI) antipsychotic medications—medications administered via intramuscular injection releasing active medication slowly into the bloodstream, yielding therapeutic blood levels for 2 weeks after each injection, and in recent formulations for 4 weeks or even 6 months [11]. LAI medications are thought to reduce medication non-adherence but meta-analyses have yielded mixed results on the effectiveness of these medications compared to oral drugs [12, 13] albeit with more positive results in a recent meta-analysis, which highlighted pre-post studies that claimed to better reflect the real-world practices of prescribing LAI antipsychotics [14].

Beginning in the 1990s, a new type of antipsychotic medication, second-generation antipsychotics (SGAs), emerged which had fewer neurological side effects than previously approved drugs, i.e., first-generation antipsychotics (FGAs). It was hoped they would thereby lead to greater acceptance and more consistent use of these medications, but, in fact also had mixed results [15, 16]. It was only in 2003 that the first LAI SGA, LAI risperidone (Risperdal Consta®) became available, combining the benefits of LAI administration with the reduced neurological side effects of SGAs. As only 11% of diagnostically appropriate VA patients [17] and 10–20% of non-VA patients [18] were prescribed LAI medications, it was of substantial importance to evaluate the potential benefits of this new treatment. In 2005, the VA CSP implemented a multisite study comparing LAI risperidone to providers’ choice of oral antipsychotic medication (CSP#555) to evaluate these potential benefits before the drug entered widespread use. Results reported 6 years later found no significant reductions in hospitalization rates (the primary outcome) after a year of LAI risperidone treatment and no differences in secondary outcomes including schizophrenia symptoms, major side effects, quality of life, or other health outcomes [19]. The study was thus designed to address a critical question that has not been well studied: did this costly treatment merit widespread use?

The current study sought to trace the impact of CSP#555, an important null study, on VA clinical practice by using national VHA administrative data to compare trends in the use of LAI risperidone and other types of antipsychotic medications by Veterans diagnosed with schizophrenia or schizoaffective disorder during the 4 years (2007–2010) before the publication of CSP#555 in 2011 and the 4 years after (2012–2015). As the paper was published in early March 2011, we focused on differences in the years before and after 2011 in the annual proportions of Veterans prescribed LAI risperidone for the first time (new starts) and in the proportions of Veterans receiving LAI risperidone at all (any use) using multivariate analysis to control for potentially confounding sociodemographic and clinical characteristics. To understand contextual factors, we also examined changes in the use of other antipsychotics during these years including both LAI and oral medications, as well as changes in VA expenditures for LAI risperidone and other antipsychotics in this population. The original protocol noted that a null result could justify systemwide discouraging of LAI risperidone use in VHA as it was many times more expensive than other approved antipsychotic medications [20]. Accordingly, we hypothesized that in the years following the publication of the trial use of LAI risperidone would decline and be associated with reduced costs for LAI risperidone and for antipsychotics generally in the VHA.

## Methods

### Design and sample

Data used in the present longitudinal observational analysis were derived from the national VHA electronic health records (EHRs) captured by the VA Cooperate Data Warehouse, which document sociodemographic, inpatient and outpatient services use, clinical diagnoses, and VA prescription fills for all Veterans treated by VHA clinics.

The study population included all VHA patients who received an ICD-9 diagnosis of schizophrenia or schizoaffective disorder (295.00 to 295.95) in an inpatient discharge abstract or outpatient clinic visit in the years 2007–2015. Veterans were represented as a unique observation for each year in which they received any antipsychotic medication regardless of whether they received a diagnosis of schizophrenia or schizoaffective disorder in that particular year.

### Measures

The VA outpatient pharmacy records were used to identify all prescriptions for antipsychotic medications from the study population. Measures were constructed to identify each year in which a Veteran had a new start on LAI risperidone and on other LAI SGAs, i.e., a year in which they had not filled a prescription for that medication in the previous year. New starts were thus defined as the first year a prescription for LAI risperidone or other LAI SGAs was filled between 2007 and 2015 and were assumed to represent physician decision-making based on current knowledge, i.e., unaffected by legacy prescription decisions. Measures were also constructed representing years in which any annual LAI risperidone prescription fills were recorded, i.e., not necessarily the first, as well as prescriptions for (1) other LAI SGAs, (2) LAI FGAs, and (3) FGA and SGA oral antipsychotics. These analyses incorporate legacy prescription decisions, less likely to be influenced by recent research.

Costs of medications to VHA (at negotiated discount prices) are recorded for each prescription in VA pharmacy benefit records. Measures were thus constructed representing annual costs per Veteran for LAI risperidone for each year, as well as for the other classes of antipsychotics noted above: LAI SGAs, LAI FGAs, and FGA and SGA oral medications. The proportion of average costs attributable to LAI SGAs was also calculated for the entire population for each year.

Sociodemographic characteristics derived from VHA EHR, included age, gender, race, marital status, and VA service-connected disability status. Comorbid psychiatric diagnoses clustered into 12 groups included alcohol abuse or dependence (ICD-9 303.xx or 305.00), drug abuse or dependence (292.01, 292.99, 304.xx, or 305.20–305.99), other psychoses (297.xx-299.xx), bipolar disorder (296.0x, 296.1x, or 296.40–296.89), major affective disorder (296.2–296.39), dysthymia (300.4x, 296.9x, 311.xx, 301.10–301.19), posttraumatic stress disorder (309.81), anxiety disorders (300.xx *excluding* 300.4x), adjustment disorder (309.xx excluding 309.81), personality disorder (301.0x), and any other psychiatric disorder excluding the above (290.00–312.99 *excluding* 305.1). Traumatic brain injury was identified using detailed codes published elsewhere [21].

VHA service use was represented by the average number of annual outpatient mental health visits for each Veteran (VA clinic stop codes 500–599), the presence of a discharge abstract for a hospitalization with a diagnostic code for schizophrenia or schizoaffective disorder, and participation in specialized VA mental health intensive case management programs, designed to treat Veterans with the most severe of mental illnesses [22].

### Analysis plan

Descriptive data on Veteran characteristics and antipsychotic medication use are presented for all unique Veterans in the study sample from 2007 to 2015. Those with data from 2007 to 2010 and those with data from 2012 to 2015 are compared with those from the reference year, 2011. Random effects mixed models were used to adjust statistical comparisons for the correlatedness of data from individuals who were included in both time periods.

**Fig. 1:**
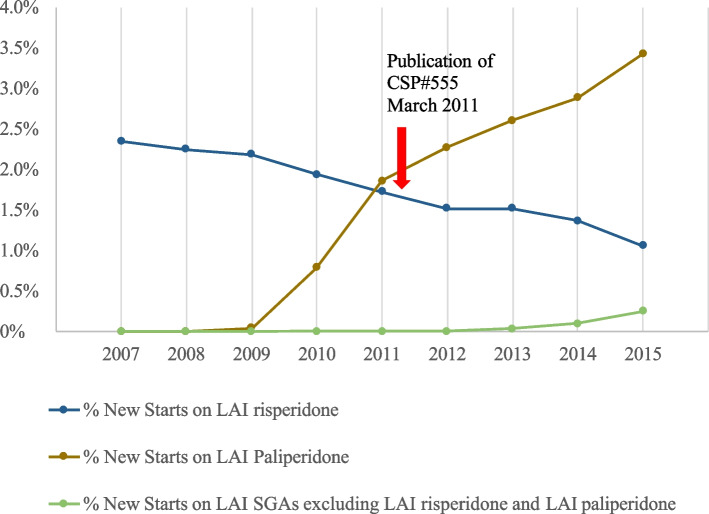
Proportion of new starts on LAI risperidone, LAI paliperidone, and other LAI SGAs: 2007–2015

The proportion of Veterans in the study sample with new starts of LAI risperidone, the primary outcome measure for this study, and new starts on other LAI SGAs are then presented graphically from 2007 to 2015 (Fig. 1).

Examination of the statistical significance of differences in the likelihood of receiving a new start on LAI risperidone as well as other LAI SGAs over the years was conducted through a random effects mixed model analysis in which the dependent variable was a dichotomous variable representing a new start on LAI risperidone among all Veterans in the population seen that year, and the independent variables were the calendar years 2007–2010 and 2012–2015 with the year (2011) in which CSP#555 was published [19] as the reference year (marked with an asterisk on the tables). Potentially confounding sociodemographic and diagnostic covariates (described earlier), representing possible confounding changes in the characteristics of the study population from year to year, were added through a stepwise procedure with forward selection. While these analyses identified significant differences in new starts on LAI risperidone for each year, as compared to 2011 (with significance marked by “^” on the tables), a more detailed evaluation of the statistical significance of differences between adjacent pairs of years was determined by comparing the odds ratio (OR) for any given year with the 95% confidence interval (CI) of the OR from the year before (with significance marked by “#” on the tables). If the OR for a given year was outside of the 95% CI of the previous year, it represented a statistically significant difference.

In addition, interrupted time series analysis was used to determine whether the slope of the curve (Fig. 1) reflecting a decline in new starts of LAI risperidone in the years 2012–2015, following publication, was more steeply negative than in the years 2007–2010.

Similar analyses were conducted for new starts on other LAI SGAs and on all LAI SGAs to provide context for understanding changes in prescribing for LAI risperidone.

An additional set of analyses examined the proportion of Veterans specifically prescribed LAI risperidone in each year who switched to LAI paliperidone (Invega®; a 4-week LAI SGA available after 2009) in the next year.

These analyses were repeated for *any* use of LAI risperidone, i.e., not just first starts, and other antipsychotics to assess changes and differences in the ongoing use of LAI risperidone and other antipsychotics.

Total annual VHA expenditure data were examined through random effects mixed models that compared average expenditures for LAI risperidone and other antipsychotics in each year to the reference year (2011), again adjusting for potential confounders. Expenditures for LAI risperidone were compared across the years, again with 2011 as the reference year. Costs for other LAI SGAs per Veteran were also examined along with the proportion of all antipsychotic expenditures attributable to LAI SGAs to determine the total budgetary impact of the observed changes on prescribed medications.

## Results

### Sample

In the total sample of 115,172 Veterans, 74,007 (64.3%) were seen in both periods (2007–2011 and 2012–2015); 21,768 (18.9%) were seen exclusively from 2007 to 2011; and 19,397 (16.8%) exclusively from 2012 to 2015.

Unique Veterans in the sample averaged 56 years of age with 7.7% women, 26.3% receiving VA service-connected disability payments, 13.1% seen in the intensive case management programs, and 29.9% hospitalized for schizophrenia during the study period. They had an average of 29.4 mental health outpatient visits/year and 71.9% had at least one other psychiatric comorbidity.

Due to the large sample size, virtually all comparisons between periods were statistically significant (*p* < 0.0001) but none of the effect size differences would be considered of meaningful magnitude (OR > 1.5 or Cohen’s *d* > 0.20) (Ferguson, 2009, Cohen 1988; Table 1).

**Table 1:** Sociodemographics and psychiatric comorbidities of Veterans receiving antipsychotic medication for schizophrenia or schizoaffective disorder 2007–2015

**Demographics**		**All years:** **2007–2015** ***N* = 115,172**	**Pre-publication: 2007–2011** ***N* = 95,775**	**Post-publication: 2012–2015** ***N* = 93,404**	**OR/Cohen’s *d***^a^ **(95% CI)**	***p*-value**
Race/ethnicity	Black Hispanic	806 (0.7)	573 (0.6)	646 (0.7)		< .0001
	Black not Hispanic	39,373 (32.9)	29,182 (32.4)	31,434 (33.7)		
	White Hispanic	7093 (5.9)	5277 (5.9)	5816 (6.2)		
	White not Hispanic	58,693 (49.1)	44,603 (49.5)	46,456 (49.7)		
	Other Hispanic	13,26 (1.1)	892 (1.0)	1061 (1.1)		
	Other not Hispanic	12,274 (10.3)	9567 (10.6)	7991 (8.6)		
Marital status	Divorced	35,956 (30.1)	27,080 (30.1)	27,881 (29.9)		< .0001
	Married	26,703 (22.3)	20,126 (22.3)	21,102 (22.6)		
	Never	44,810 (37.5)	33,988 (37.7)	35,394 (37.9)		
	Others	12,096 (10.1)	8900 (9.9)	9027 (9.7)		
Age	Mean (SD)	56.4 (11.6)	55.3 (10.9)	57.1 (11.9)	0.164* (0.159–0.169)	< .0001
Female	*N* (%)	9348 (7.8)	6303 (7.0)	7656 (8.2)	1.19 (1.17, 1.21)	< .0001
VA service-connected disability rate	≥ 50%	30,542 (25.5)	26,528 (29.4)	23,317 (25.0)	0.80 (0.79, 0.81)	< .0001
	< 50%	6315 (5.3)	4807 (5.3)	4480 (4.8)	0.89 (0.87, 0.92)	< .0001
Participated in mental health intensive case management program	Ever in 2007**–**2015	15,134 (12.7)	9615 (10.7)	10,831 (11.6)	1.10 (1.08, 1.12)	< .0001
VA mental clinic visits (stop code 500–599)	Annualized mean (SD)	24.6 (37.7)	28.0 (47.2)	31.6 (47.1)	0.078* (0.069, 0.087)	< .0001
Deaths	*N* (%)	17,758 (14.9)	5628 (6.3)	7269 (7.8)	1.27 (1.22, 1.31)	< .0001
Inpatient diagnosis of schizophrenia or schizoaffective		34,534 (28.9)	19,639 (21.8)	18,304 (19.6)	0.87 (0.86, 0.89)	< .0001
History of mental health disorders	Alcohol use disorder	47,333 (39.6)	35,753 (39.7)	38,461 (41.2)	1.06 (1.05, 1.07)	< .0001
	Drug use disorder	48,786 (40.8)	36,107 (40.1)	39,903 (42.7)	1.12 (1.10, 1.13)	< .0001
	Substance use disorder	61,346 (51.3)	45,870 (50.9)	49,828 (53.3)	1.10 (1.09, 1.11)	< .0001
	Other mental health diagnoses	11,475 (9.6)	8196 (9.1)	9631 (10.3)	1.15 (1.13, 1.17)	< .0001
	Bipolar	36,863 (30.8)	27,732 (30.8)	30,786 (33.0)	1.11 (1.10, 1.12)	< .0001
	Major	34,782 (29.1)	24,744 (27.5)	28,525 (30.5)	1.16 (1.15, 1.17)	< .0001
	Dysthymia	65,828 (55.1)	47,903 (53.2)	53,638 (57.4)	1.19 (1.18, 1.20)	< .0001
	PTSD	37,649 (31.5)	27,041 (30.0)	30,923 (33.1)	1.15 (1.14, 1.17)	< .0001
	Anxiety	49,023 (41.0)	35,486 (39.4)	40,553 (43.4)	1.18 (1.17, 1.19)	< .0001
	Adjustment	21,980 (18.4)	15,125 (16.8)	17,803 (19.1)	1.17 (1.15, 1.18)	< .0001
	Personality	22,321 (18.7)	16,729 (18.6)	18,208 (19.5)	1.06 (1.05, 1.07)	< .0001
	Any psychiatric comorbidity	114,924 (96.1)	87,587 (97.2)	91,401 (97.9)	1.31 (1.26, 1.35)	< .0001
	Alzheimer’s	2017 (1.7)	1653 (1.8)	1274 (1.4)	0.76 (0.74, 0.78)	< .0001
	Dementia	6562 (5.5)	5417 (6.0)	4330 (4.6)	0.74 (0.71, 0.77)	< .0001
	Traumatic brain injury	7966 (6.7)	5417 (6.0)	6742 (7.2)	1.22 (1.19, 1.24)	< .0001
	More than 1 comorbidity listed above	84,916 (71.0)	62,911 (69.8)	68,782 (73.6)	1.21 (1.19, 1.22)	< .0001

^a^Reported as Cohen’s *d*

### Changes in prescribed medications

The proportion of Veterans diagnosed with schizophrenia or schizoaffective disorder who received new, first-time, prescriptions for LAI risperidone decreased steadily from 2007 to 2015 (Fig. 1), even before the publication of CSP-555. In contrast, the proportion of Veterans receiving new starts on LAI paliperidone increased dramatically after its introduction from 2009 to 2015 (Fig. 1) while new starts on other LAI SGAs increased to a negligible degree from 2013 to 2015.

Statistical analyses showed that after adjusting for other Veteran characteristics, there were a significantly greater proportion of new starts on LAI risperidone from 2007 to 2009 than in 2011 (Table 2, marked by “^”), reflecting a decline in new starts on LAI risperidone that preceded the publication of CSP#555. The first year in which there was a statistically significant decline in new starts on LAI risperidone from the prior period was 2009–2010 (Table 2, marked by “#”). New starts on LAI risperidone continued to fall after 2011, becoming significantly different from 2011 in 2014 and 2015, several years following the publication of CSP#555.

**Table 2:** New starts on LAI SGAs among study sample: 2007–2015 with comparison to 2011

Year	Patients with any antipsychotics	New starts on LAI risperidone		New starts on LAI paliperidone		New starts on SGA LAIs (excluding LAI risperidone and paliperidone)^+^		New starts on all LAI SGAs	
		***N***	**%**	***N***	**%**	***N***	**%**	***N***	**%**
2007	67,023	1574	2.3^^^	0	0	0	0	1574	2.3^^^
2008	68,616	1542	2.2^^^	0	0	0	0	1542	2.2^^,#^
2009	70,468	1543	2.2^^^	30	0^^^	0	0	1573	2.2^^,#^
2010	71,346	1383	1.9^#^	560	0.8^^,#^	1	0	1944	2.7^^,#^
2011*	72,000	1244	1.7	1338	1.9	1	0	2583	3.6
2012	72,410	1102	1.5	1645	2.3^^^	3	0	2750	3.8^^^
2013	72,535	1100	1.5	1890	2.6^^,#^	23	0	3013	4.2^^,#^
2014	72,371	988	1.4^^^	2090	2.9^^,#^	73	0.1	3151	4.4^^,#^
2015	73,008	771	1.1^^,#^	2503	3.4^^,#^	180	0.2	3454	4.7^^,#^
**All years**	**115,172**	**11,929**	**10.0%**	**13,229**	**11.1%**	**566**	**0.5%**	**25,724**	**21.5%**
**Change**	**5985**	** − 803**	** − 1.2%**	**2503**	**3.4%**	**180**	**0.25%**	**1880**	**2.4%**
**Percent change**	**8.9%**	** − 51.0%**	** − 52.2%**	**–-**	**–-**	**–-**	**–-**	**119.4%**	**104.3%**

^*^Used as the reference year to define pre- and post-publication of a cooperative study (CSP#555)

^^^Statistically significant difference as compared to the reference year

^#^Statistically significant difference when compared to the previous year (excluding comparisons with the reference year)

^+^Sample was too small to conduct a statistical model

In the interrupted time series analysis, the *p*-value for the interruption slope term was 0.70, a non-significant value. There was thus no change in the declining slope after publication (Fig. 1). The intercept of the interruption term also was insignificant.

In contrast, new starts on another LAI SGA, LAI paliperidone, once it became available to VHA in 2009, increased every year thereafter to a total of 3.4% of the entire study population by 2015 (Table 2). The intersection between paliperidone and risperidone prescription thus occurs before the publication of CSP#555. New starts on other LAI SGAs increased after 2011, but by 2015 only involved 0.2% of the sample. New starts on any LAI SGA increased steadily and significantly after 2009 to 4.7% of the sample (Table 2, last column), predominantly representing growth in the use of LAI paliperidone.

The proportion of specific switches from LAI risperidone to LAI paliperidone doubled from 5.9% in 2009–2010 to 13.3% in 2010 to 2011 and remained at more than 1.5 times the 2009–2010 rate of switching through 2015 (Table 3, last column).

**Table 3:** Proportion of patients prescribed LAI risperidone who switched to LAI paliperidone in the subsequent year

Index year	Prior year	Prescribed LAI risperidone in prior year	Prescribed LAI paliperidone in subsequent year	Switching over to paliperidone from risperidone (%)	OR (95% CI)	Risk ratio (subsequent year changes compared to 2009–2015 change)
2010	2009	4361	255	5.9^^^	0.40 (0.34, 0.47)	
2011*	2010	4344	578	13.3		2.28
2012	2011	4098	495	12.1	0.90 (0.79, 1.03)	2.06
2013	2012	3760	370	9.8^^,#^	0.73 (0.63, 0.83)	1.68
2014	2013	3601	384	10.7^^^	0.80 (0.70, 0.92)	1.82
2015	2014	3223	403	12.5	0.97 (0.84, 1.11)	2.14

^*^Used as the reference year to define pre- and post-publication of a cooperative study (CSP#555)

^^^Statistically significant difference as compared to the reference year

^#^Statistically significant difference when compared to the previous year (excluding comparisons with the reference year)

Turning to all LAI risperidone use (not just new starts), the peak year for both the total number of Veterans prescribed LAI risperidone and the proportion of all Veterans in the sample prescribed LAI risperidone occurred in 2009, 2 years *before* the publication of CSP#555, and declined significantly (*p* < 0.0001) every year after that, and by a total of 21.7% from 2007 to 2015 (Table 4). As with new starts, the number and proportion of all patients prescribed LAI paliperidone increased every year after it became available in 2009 as did the proportion of all patients on any LAI SGA, which peaked at 8.99% in 2015. While Veterans prescribed LAI SGAs increased by 80.5% from 2007 to 2015, Veterans prescribed oral SGA medications increased by only 11.4%. In contrast, the proportions of Veterans on FGAs, including both LAI FGAs and oral FGAs, declined by 24.6% and 3.0% respectively (Table 4).

**Table 4:** Numbers of patients diagnosed with schizophrenia or schizoaffective disorder prescribed LAI antipsychotics from 2007 to 2016*

Year	Patients with any antipsychotics	LAI risperidone		Other LAI SGA	LAI paliperidone		Any LAI SGA		Any FGA LAI	Oral SGA	Oral FGA
		***N***	**%**	***N***	***N***	**%**	***N***	**%**	***N***	***N***	***N***
2007	66,985	3625	5.4^^^	–-	–-	–-	3625	5.4^^^	5499	39,975	35,704
2008	68,565	3914	5.7^^,#^	–-	–-	–-	3914	5.7^^,#^	5361	41,773	35,372
2009	70,401	4258	6.0^#^	29	29	0.04^^^	4268	6.1^^,#^	5398	43,002	36,005
2010	71,250	4252	6.0^^^	512	511	0.7^^,#^	4487	6.3^^,#^	5237	43,335	36,637
2011*	71,842	4024	5.6	1346	1345	1.9	5036	7.0	5061	42,986	37,032
2012	72,230	3723	5.2^^^	1949	1944	2.7^^^	5431	7.5^^^	4855	42,617	36,976
2013	72,275	3541	4.9^^,#^	2585	2547	3.5^^,#^	5889	8.2^^,#^	4535	42,669	37,266
2014	71,959	3199	4.5^^,#^	3116	2914	4.1^^,#^	6075	8.4^^,#^	4133	43,226	35,889
2015	72,777	2838	3.9^^,#^	3991	3564	4.9^^,#^	6542	9.0^^,#^	4145	44,531	34,617
**Change**	**5792**	** − 787**	** − 1.5%**	**3991**	**3564**	**4.9%**	**2917**	**3.6%**	** − 1354**	**4556**	** − 1087**
**%Change**	**8.6%**	** − 21.7%**	**27.9%**	**–-**	**–-**	**–-**	**80.5%**	**66.1%**	** − 24.6%**	**11.4%**	** − 3.0%**

^*^Used as the reference year to define pre- and post-publication of a cooperative study (CSP#555)

^^^Statistically significant difference as compared to the reference year

^#^Statistically significant difference when compared to the previous year (excluding comparisons with the reference year)

As hypothesized, annual VHA expenditures per Veteran on LAI risperidone increased from 2007 to 2009 but began to decline in 2010, before CSP#555 was published, and especially in 2011, the year of publication of CSP#555 (Table 5). LAI risperidone costs per Veteran continued to decline sharply every year thereafter (2012–2015).

**Table 5:** Annual antipsychotic medication costs among patients with schizophrenia or schizoaffective disorder

Year	All antipsychotics	LAI risperidone	All LAI SGAs	LAI paliperidone	LAI FGAs	SGA oral	FGA oral	LAI SGA expenditures as % of total antipsychotic expenditures per Veteran
2007	$122,170,267	$12,914,274^^^	$12,914,274^^^	$0	$371,238	$76,078,149	$32,806,605	10.6^^^
2008	$128,140,495	$15,085,187^^^	$15,085,187^^,#^	$0	$348,750	$82,396,607	$30,309,950	11.8^^^
2009	$115,066,407	$16,832,583	$16,881,109^^^	$48,526^^^	$358,936	$86,271,426	$11,554,935	14.7^^,#^
2010	$116,571,834	$16,878,621	18,142,921^^,#^	$1,263,172^^,#^	$370,713	$91,405,234	$6,652,966	15.6^^,#^
2011*	$117,202,917	$16,062,864	$20,802,052	$4,737,495	$805,706	$90,968,967	$4,626,193	17.8
2012	$96,742,862	$14,562,180	$22,761,616^^^	$8,192,594^^^	$1,017,493	$68,812,359	$4,151,395	23.5^^^
2013	$76,508,388	$12,757,391	$23,476,864^^^	$10,544,810^^^	$846,056	$48,876,894	$3,308,575	30.7^^,#^
2014	$70,692,177	$12,378,899	$26,402,932^^,#^	$13,341,523^^,#^	$919,701	$40,500,060	$2,869,484	37.4^^^
2015	$73,982,381	$11,135,327^^^	$30,164,815^^,#^	$17,156,228^^^	$869,519	$40,245,255	$2,702,792	40.8^^,#^
**Change**	**($48,187,886)**	**($1,778,947)**	**$17,250,541**	**$17,156,228**	**$498,281**	**($35,832,894)**	**($30,103,814)**	**30.2%**
**Percent change**	** − 39.4%**	** − 13.8%**	**133.6%**	–-	**134.2%**	** − 47.1%**	** − 91.8%**	**285.7%**

^*^Used as the reference year to define pre- and post-publication of a cooperative study (CSP#555)

^^^Statistically significant difference as compared to the reference year

^#^Statistically significant difference when compared to the previous year (excluding comparisons with the reference year)

In 2011 and 2012, two of the most widely used oral SGAs, olanzapine and quetiapine, lost their patent protection and total VA expenditures on oral SGAs dropped substantially by 47.1% from 2017 to 2015 (Table 5, column 7). Expenditures on oral FGAs also declined by 92% over the same period (Table 5, column 8). Total antipsychotic costs per Veteran declined by 39.44% 2007–2015 (Table 5, column 2), only 3.6% of which was attributable to reduced per-patient expenditures on LAI risperidone. This overall change reflected the offsetting effect of increased expenditures on all LAI SGAs of $17.2 million (99% of which was for increased expenditures on LAI paliperidone) and by a reduction of $65.9 million on expenditures for oral SGA and FGA medications (Table 5, sum of columns 7 and 8). As expenditures on LAI risperidone declined, expenditures on LAI paliperidone increased substantially after its introduction in 2009, as did per Veteran expenditures on all LAI SGAs combined (Table 5, column 4).

As a result of the large changes in per Veteran expenditures on oral medication, total VHA expenditures on antipsychotics for this population declined by $48 million (39%, Table 5, column 2), while the proportion of all antipsychotic expenditures on LAI SGAs, (predominantly paliperidone) increased from 10 to 40% of all antipsychotic expenditures (Table 5, final column). In sharp contrast to changes in expenditures, the proportion of individual Veterans on LAI SGAs only increased from 5.4 to 8.99% between 2007 and 2015 (Table 3, column 9).

## Discussion

As hypothesized, new starts on LAI risperidone in VHA, as well as its total annual use and related expenditures declined following the publication of a multisite randomized clinical trial of the medicine with null results, thus failing to provide justification for use of this medication. The policy option of discouraging its use had, in fact, been identified in the trial protocol [20] as a justifiable response to null study results, although no such policy has been implemented.

However, further examination of data from the years *prior* to the publication of CSP#555 showed the decline in new starts of LAI risperidone preceded the publication of CSP#555 and thus could not be entirely attributed to the study. We also did not find evidence that the decline in new starts on LAI risperidone proceeded at an accelerated rate after the publication of CSP#555.

The declining use of LAI risperidone seems largely to coincide with the simultaneous and rapid increase in prescriptions for LAI paliperidone during the 2 years prior to the publication of CSP#555. However, the sharp increase in *specific* switches from LAI risperidone to LAI paliperidone during and after the year CSP#555 was published could possibly represent a specific clinician response to null study findings. The declining use of LAI risperidone primarily coincides with clinician choices favoring a new 4-week LAI medication, paliperidone, the active metabolite of risperidone, and manufactured by the same company.

Perhaps the most striking feature of these results is that they could not have been anticipated in 2005 when CSP#555 was designed. What was known was only that medication non-adherence was a determinant of relapse and rehospitalization in schizophrenia and that LAI medication improved compliance. There was far more limited evidence that LAI antipsychotics reduced relapse or improved symptom outcomes. Second-generation antipsychotics appeared to be better tolerated than conventional medications [14] and the combination of LAI delivery and SGA tolerability was promising and in need of evaluation.

Although CSP#555 offered no support for the value of LAI risperidone, or, by implication, for LAI SGAs more generally, it seems likely that the rapidly expanded use of LAI paliperidone reflected the greater face-value of a 4-week as contrasted to a 2-week LAI medication and vigorous marketing of this new but only modestly different treatment whose superiority to LAI risperidone or any other treatment had not been demonstrated.

The scientific questions posed by CSP#555, in fact, remained unresolved. A RCT quite similar to CSP#555, published around the same time, also found no difference in relapse or rehospitalization between LAI risperidone and oral antipsychotics [23] and a second RCT comparing LAI paliperidone and LAI haloperidol [24] found no clinical advantage for LAI paliperidone, while a secondary analysis of VHA data found that LAI haloperidol was the more cost-effective treatment [25]. In contrast, an observational study examined VHA administrative data and reported cost-savings with paliperidone as compared to oral medications, albeit without random assignment [26].

The most recent meta-analysis of 137 studies, published 10 years after CSP#555 [14], found significant but small effects in both randomized trials (RR = 0.88 for relapse in 29 studies of 7833, *p* = 0.03) and cohort studies (RR = 0.92 in 44 studies of 106,136 patients, *p* = 0.004). More substantial effects were found only in studies with weak pre-post designs (RR = 0.44 in 28 studies of 17,876 patients, *p* < 0.0001).

Perhaps the strongest evidence favoring LAIs was published in 2020 from a 489-patient cluster randomized trial showing less risk of hospitalization or relapse among patients with early episode schizophrenia at sites randomized to encourage the use of LAI aripiprazole as compared to sites that provided usual care. This more supportive evidence, published 9 years after CSP#555, is of unknown generalizability to adults with long-term illness such as those treated by VHA. Thus, VHA prescribers increasingly used LAI SGAs in the years after 2009, in spite of the null findings from CSP#555 or positive evidence from other studies.

The discrepancy between empirical findings and subsequent changes in clinical practice observed in this study highlights the role of unanticipated non-research commercial factors in shaping the impact of major effectiveness studies. A similarly paradoxical result was documented in response to the NIMH-funded CATIE study of 1432 patients diagnosed with schizophrenia that compared several oral SGAs to each other [15]. Following its publication, use of the drug that did best on the primary effectiveness outcome of the study (olanzapine) actually declined in clinical practice, most likely due to increasing concern about its metabolic side effects, and due to the approval of a new actively marketed drug with fewer metabolic side effects (aripiprazole), not evaluated in CATIE, that showed substantially increased use, also without evidence of its superiority [27]. VA cooperative study #504 evaluating oral risperidone for PTSD (CSP#504) also reported unexpected null results in 2011 [28]. A subsequent national study of VHA pharmacotherapy for PTSD from 2008 to 2018 showed an annual decline of about 8.3% in risperidone use before CSP#504 publication with a 7.4% annual decline (virtually unchanged) over the next 7 years. Here, again no impact of the trial was observed but rather a persistent decline in risperidone use which also appeared to have been replaced with the new actively marketed drug, aripiprazole, albeit without evidence of its effectiveness in PTSD [29].

In contrast to CSP#555, some trials quickly lead to major changes in practice. As soon as recent trials demonstrated that COVID-19 vaccines were highly effective, they received regulatory approval and major efforts were initiated to foster their widespread use [30]. Similarly, once a major trial clearly revealed the risk of cardiac mortality with rofecoxib [31], the drug was withdrawn from the market altogether. These examples point to a major factor that is likely to affect the impact of individual RCTs—the magnitude of effects and seriousness of the health benefits or harms they demonstrate. CSP#555 (as well as CSP#504) found virtually no positive or negative effect and this may account for its limited impact. Another influential factor is whether a study leads to explicit policy changes or directives by governmental agencies as in the case of the FDA and CDC promoting COVID-19 vaccines.

### Limitations

The primary limitation of this study is that temporal trends in VHA administrative data do not allow causal conclusions about the impact of CSP#555 since no information is available from a “control” healthcare system with no exposure to the results of the study. Nor are data available on the extent to which clinicians or patients and their families were aware of and understood the implications of CSP#555, or on either their exposure or response to other studies of LAI antipsychotics, their medication preference, their involvement with key opinion leaders, or exposure to marketing initiatives. In addition, this study addressed only broad national trends and not small area variations in practice changes. Finally, it examines the impact of only one publication, and changes in practice may only emerge in response to multiple consistent studies and meta-analyses.

### Policy implications

This discussion highlights the importance of designing practical clinical trials, of critically considering their potential effect size and health impact, and of possible future of prescribing, research, and marketing contexts. Protocols may be enhanced by including a plan for readily adapting their design/methods should new and relevant developments emerge during the course of the trial. Planned dissemination and policy implementation efforts should also be part of the study design, as is increasingly the case in the VA Cooperative Studies Program.

## Conclusion

The declining use of LAI risperidone after the publication of null findings from CSP#555 likely reflected the expanded use of LAI paliperidone more than study results, although the declining use of LAI risperidone may have been reinforced by null study findings. Unanticipated changes in the therapeutic environment can complicate the implementation of clinical trial results and deserve further attention.

## Data Availability

Veterans Health Administration data used for these analyses are not available to the public.

## Abbreviations

CSP: Cooperative Studies Program
CI: Confidence interval
EHR: Electronic health record
FDA: Food and Drug Administration
FGA: First-generation antipsychotic
LAI: Long-acting injectable
OR: Odds ratio
RCT: Randomized clinical trial
SGA: Second-generation antipsychotic
VA: Veterans Affairs
VHA: Veterans Health Administration
